# Quantitative evaluation and comparison of coronary artery characteristics by 3D coronary volume reconstruction

**DOI:** 10.1038/s41598-020-80928-4

**Published:** 2021-01-13

**Authors:** Yongcheol Kim, Jonathan James Hyett Bray, Benjamin Waterhouse, Alexander Gall, Georgia May Connolly, Eva Sammut, Vito Domenico Bruno, Robert Tulloh, David Adlam, Thomas W. Johnson

**Affiliations:** 1 Division of Cardiology, Department of Internal Medicine, Yonsei University College of Medicine and Cardiovascular Center, Yongin Severance Hospital, Yongin, Republic of Korea; 2 Bristol Heart Institute, University Hospitals Bristol NHS Foundation Trust, Upper Maudlin Street, Bristol, BS2 8HW UK; 3 University of Bristol, Bristol, UK; 4 University of Bristol Medical School – Translational Health Science, Bristol, UK; 5 Department of Cardiovascular Sciences, NIHR Leicester Biomedical Research Centre, Leicester, UK

**Keywords:** Cardiology, Interventional cardiology, Cardiovascular diseases

## Abstract

Non-atherosclerotic abnormalities of vessel calibre, aneurysm and ectasia, are challenging to quantify and are often overlooked in qualitative reporting. Utilising a novel 3-dimensional (3D) quantitative coronary angiography (QCA) application, we have evaluated the characteristics of normal, diabetic and aneurysmal or ectatic coronary arteries. We selected 131 individuals under 50 years-of-age, who had undergone coronary angiography for suspected myocardial ischaemia between 1st January 2011 and 31st December 2015, at the Bristol Heart Institute, Bristol, UK. This included 42 patients with angiographically normal coronary arteries, 36 diabetic patients with unobstructed coronaries, and 53 patients with abnormal coronary dilatation (aneurysm and ectasia). A total of 1105 coronary segments were analysed using QAngio XA 3D (Research Edition, Medis medical imaging systems, Leiden, The Netherlands). The combined volume of the major coronary arteries was significantly different between each group (1240 ± 476 mm^3^ diabetic group, 1646 ± 391 mm^3^ normal group, and 2072 ± 687 mm^3^ abnormal group). Moreover, the combined coronary artery volumes correlated with patient body surface area (r = 0.483, *p* < 0.01). Inter-observer variability was assessed and intraclass correlation coefficient of the total coronary artery volume demonstrated a low variability of 3D QCA (r = 0.996, *p* < 0.001). Dedicated 3D QCA facilitates reproducible coronary artery volume estimation and allows discrimination of normal and diseased vessels.

## Introduction

Aneurysmal vascular disease was first reported post-mortem over 200 years ago^1^ and coronary ectasia was first described in 1966^2^. Qualitative coronary artery dilatation is evident in up to 10% of all diagnostic coronary angiograms^3^. However, defining true aneurysm and ectasia is challenging with a lack of clearly quantifiable and reproducible cut-offs. An aneurysm is simple to define when discrete, arising in mid-vessel, and bordered by normal calibre proximal and distal segments. However, aneurysms frequently associate with atheromatous or diffusely ectatic segments and consequently defining the abnormalities is more challenging. The difficulties in effective quantification are reinforced by the myriad definitions for aneurysm and ectasia found within the literature^4^.

We have previously expressed a major concern regarding the accuracy of reporting and recognition of aneurysm/ectasia in adult coronary angiographic assessment^3^. In considering strategies to improve diagnosis it was noted that paediatric cardiologists have developed a robust method for coronary artery calibre measurement, however, paediatric and adult coronary assessments pose different challenges. In paediatric cardiology, an echocardiography-based assessment of coronary dimensions is commonly used. This assessment, usually based on visual internal diameter provides a z-score (the number of standard deviations away from the mean for a population of the same body surface area, age and sex) and is used to guide further management^5^. Assessment for aneurysm and ectasia in adult coronary arteries is not possible by echocardiography and is complicated by the potential for concomitant development of stenotic atherosclerotic disease.

Conventional 2-dimensional (2D) quantitative coronary angiography (QCA) is a highly reproducible computer-assisted technique, and is widely used to evaluate CAD in research settings^6^. However, 2D QCA has fundamental limitations as coronary angiography only provides a 2D image of the 3-dimensional (3D) structure of a coronary artery^7^. Advances in imaging software have facilitated 3D QCA, with generation of a 3D reconstruction of the coronary artery from two angiographic images, acquired at different angles^8^. This allows evaluation of vessel segment length, diameter and volume. We propose that plotting coronary volumes against a population-based normal range, with z-scoring, equivalent to the paediatric echocardiographic method, would assist in the diagnosis of aneurysm and ectasia, where the angiographic appearances are ambiguous.

As proof of principle, we have tested this new method of vessel assessment in 3 distinct populations of patient assessed by coronary angiography. In this study, we report the 3D QCA characteristics of the coronary arteries of patients with diabetes without evidence of significant coronary artery disease (CAD), in non-diabetic patients without evidence of significant CAD and in patients with angiographic evidence of aneurysmal or ectatic coronary arteries. Furthermore, we tested inter-observer variability of this novel analysis to ensure that the technique could be deployed clinically.

## Results

### Study population

From a total of 1437 patients, 131 individuals were included from three groups: 42 (normal angiography group), 36 (diabetic group), and 53 (abnormal group: 28 aneurysmal and 25 ectatic). The average age of the 131 patients was 43.2 ± 5.3 years, 76.3% were male, with a near-exclusive population of males in the abnormal group (94.3% *p* < 0.01). Differences in risk factor profile were observed between groups, with the greatest number of risk factors in the diabetic cohort (3.4 ± 1.1) and least in the normal group (0.8 ± 1.2, *p* < 0.01). The majority of patients (74.8%) had right dominant coronary anatomy. No difference in height was observed between groups (172.8 ± 10.1 cm) but variation in weight was observed (81.7 ± 17.4 kg normal, 88.4 ± 20.3 kg diabetic & 96.8 ± 29.1 kg abnormal group, *p* = 0.018), resulting in a significant difference in BMI & BSA between groups. All baseline characteristics are shown in Table S1 in the supplementary material.

### Data collected for coronary artery reconstruction by 3D QCA

3D coronary artery reconstruction was performed in a total of 1025 segments from 131 patients. In total, 23 segments were uninterpretable due to failure to generate 3D reconstruction (18 segments in 13 patients), or anatomical absence of the LMCA (5 patients). Data collected by the 3D QCA software included lumen volume, proximal and distal diameters, and segment length.

### Total coronary artery volume

**Figure 1:**
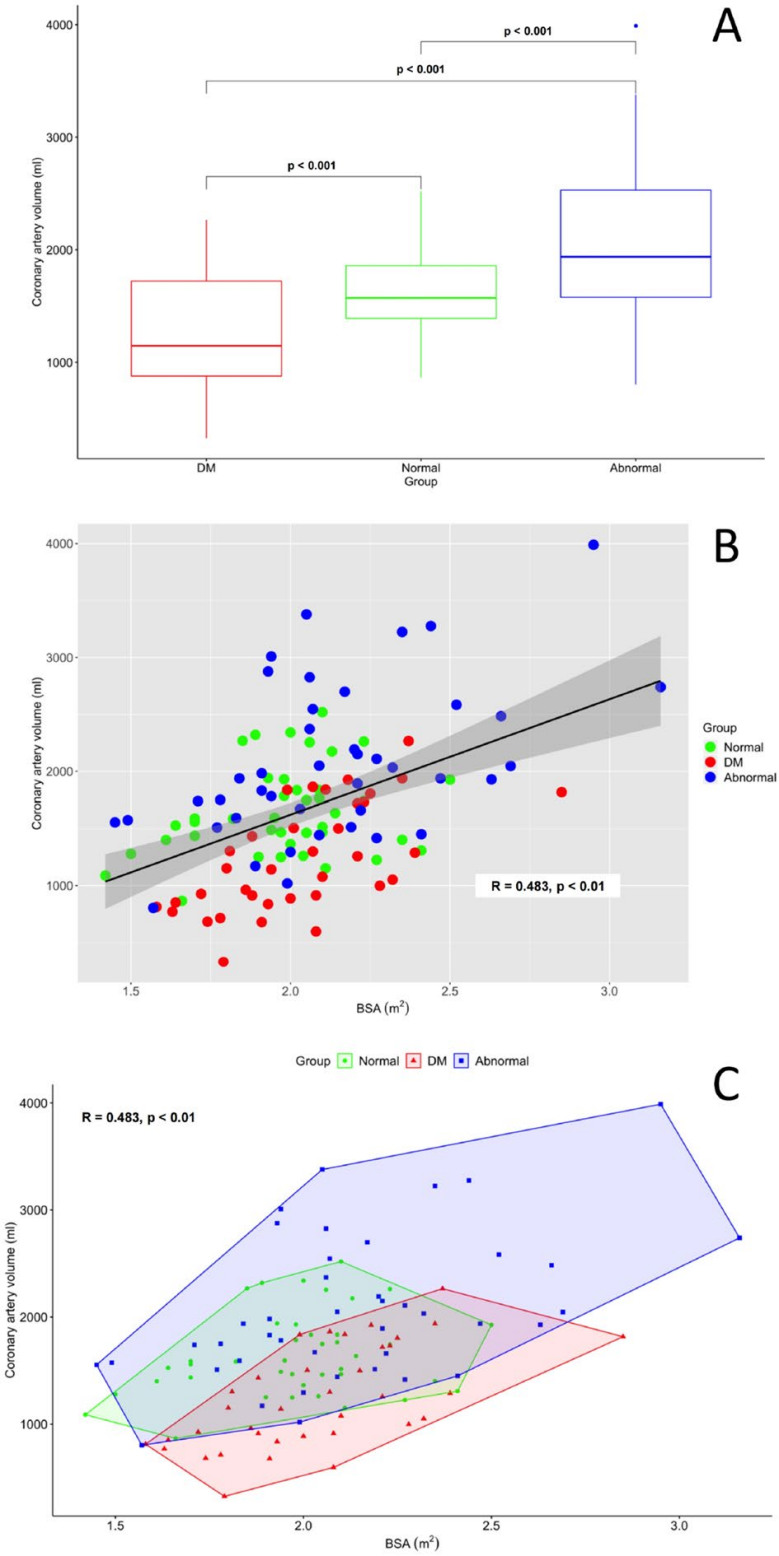
Panel **A**—The quantification of total coronary artery volume (LAD + LCX + RCA + LMCA) for three populations with non-atherosclerotic diabetic, normal, and dilated coronary disease. Panel **B**—The correlation of total coronary artery volume with body surface area (BSA m^2^) with groups identified by colour (diabetes = red; normal = green; abnormal = blue) with R = 0.48 (*p* < 0.01). Panel **C**—Total coronary artery volume and BSA correlation with clustering of groups to identify distribution within groups. Abbreviations: *BSA* body surface area, *DM* diabetes mellitus, *LAD* left anterior descending artery, *LCX* left circumflex artery, *LMCA* left main coronary artery, *RCA* right coronary artery.

The total coronary volume was defined as the combined volume of the major coronary arteries which was the summation of volumes from the RCA (segments 1, 2, and 3), LAD (segments 6 and 7), and LCX (segments 11 and 13), and LMCA (segment 5). Quantification of total coronary volume was possible in 118 (90.1%) of 131 patients, including 40 (95.2%) in the normal group, 36 (100%) in the diabetic group, and 42 (79.2%) in the abnormal group. The total coronary volume was significantly different between each group (1240 ± 476 mm^3^ diabetic group, 1646 ± 391mm^3^ normal group, and 2072 ± 687 mm^3^ abnormal group—see Fig. 1A). In addition, total coronary volume had a significant correlation with body surface area (BSA) (R = 0.483, *p* < 0.001—see Fig. 1B). The distribution of total coronary artery volume and BSA between groups is graphically represented in Fig. 1C, indicating greater variation within the abnormal group. Despite this greater variation, a significant correlation between volume and BSA was observed in the abnormal group (R = 0.493, *p* < 0.001).

The volumes of each segment, major coronary artery and combined volumes are shown in Table 1. Importantly, the proximal segments in all three major coronary territories and the LMCA of the abnormal group had consistently larger volumes compared to both the normal and diabetic groups. Additionally, the LAD and LCX volumes in the diabetic group were significantly smaller than in the normal group.

**Table 1:** Comparison of coronary volumes in groups.

	DM group (n = 36)	Normal group (n = 40)	Abnormal group (n = 42)	*p* value^§^
	Volume (mm^3^)	Volume (mm^3^)	Volume (mm^3^)	
Total coronary artery	1240 (476)*^,^^†^	1646 (391)*^,‡^	2072 (687)^†,^^‡^	< 0.001
LAD	278 (127)*^,^^†^	389 (123)*^,‡^	468 (218)^†,^^‡^	< 0.001
Proximal LAD	137 (83)*^,†^	202 (88)*^,‡^	271 (164)^†,‡^	< 0.001
Mid LAD	142 (77)^†^	187 (85)	206 (140)^†^	0.026
LCx	207 (126)*^,†^	292 (116)*	370 (237)^†^	< 0.001
Proximal LCx	97 (60)^†^	129 (77)^‡^	189 (147)^†,‡^	0.001
Mid LCx	106 (102)	164 (89)^†^	181 (134)^†^	0.011
RCA	606 (340)^†^	803 (332)	980 (493)^†^	< 0.001
Proximal RCA	241 (139)^†^	335 (173)^‡^	441 (231)^†,‡^	< 0.001
Mild RCA	146 (116)*^,†^	214 (120)*	228 (135)^†^	0.011
Distal RCA	218 (132)	254 (138)	312 (234)	0.065
LMCA	120 (81)^†^	118 (75)^‡^	179 (102)^†,‡^	0.002

Data are expressed as mean (SD).

*LAD* left anterior descending artery, *LCx* left circumflex artery, *RCA* right coronary artery, *LMCA* left main coronary artery.

*Significant difference between DM and Normal.

^§^ANOVA for DM group vs. Normal group vs. Abnormal group.

^†^Significant difference between DM and abnormal.

^‡^Significant difference between normal and abnormal.

### Data of length and lumen diameter of coronary arteries by 3D QCA

The total length of the LAD and RCA did not differ, however differences in the total length of the LCX and LMCA were observed between groups. The diabetic group had a shorter LCX length, due to a significantly shorter mid segment (CASS segment 13). Contrary to this, the diabetic group had a significantly longer LMCA compared with the normal group (10.9 ± 4.6 vs 8.2 ± 4.4 mm, *p* < 0.001), see Table S2.

Consistent with the observation that the abnormal group had greater proximal segment volumes, the segment averaged and proximal reference diameters for all measurements in the abnormal group were larger in comparison to both other groups. Segmental analysis of the diabetic group coronary lumen diameters consistently demonstrated the smallest measurements across all three groups, see Table S3. In the light of the observation of differences in segmental length between groups, an additional analysis was undertaken to correct for segment length, using a volume/length ratio (mm^3^/mm), and demonstrated a consistent difference between all 3 patient groups (diabetes < normal < abnormal), see Table S4.

### Inter-observer variability

**Figure 2:**
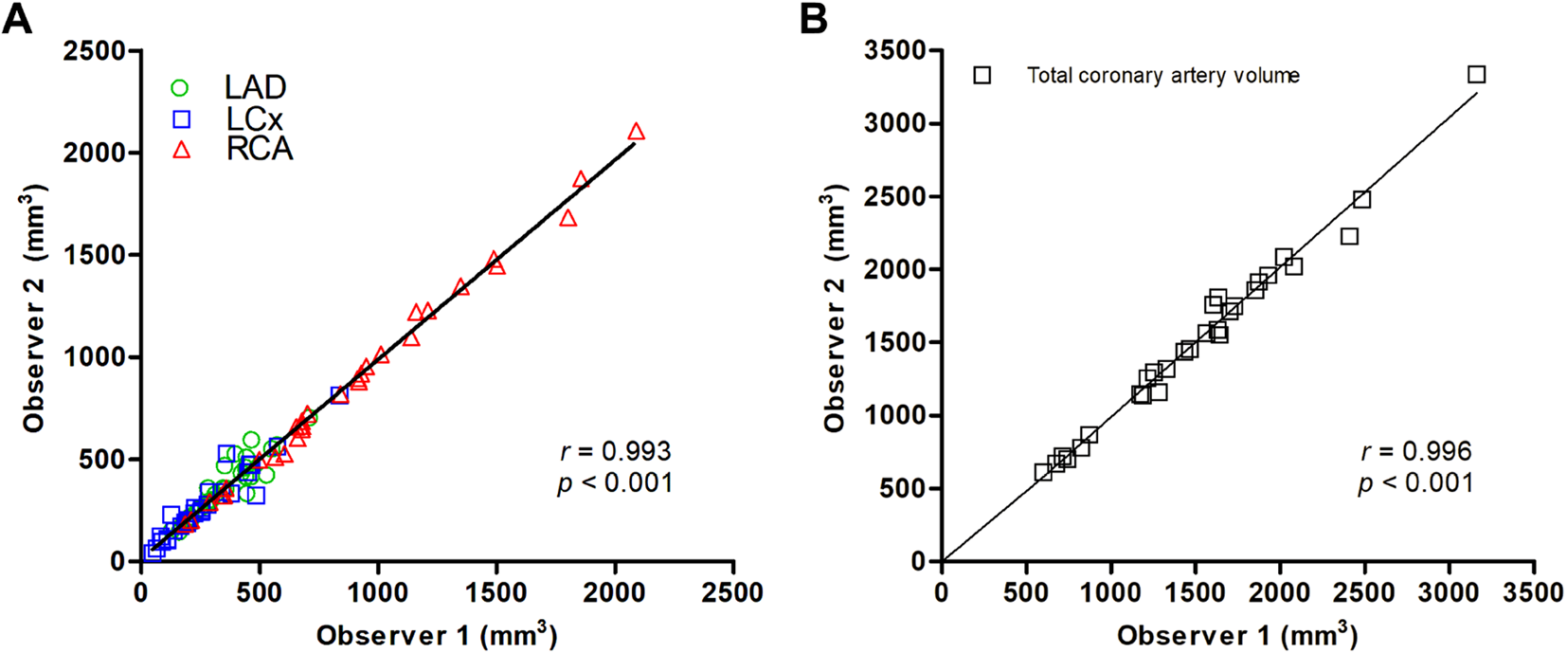
Correlation regarding the volume of 89 segments (**A**) and the total coronary volume of 29 patients (**B**) between two observers. Abbreviations: *LAD* left anterior descending artery, *LCX* left circumflex artery, *RCA* right coronary artery.

A total of 89 segments from 30 patients were randomly re-analysed by a second observer to investigate inter-observer variability of 3D QCA. This included 9 normal, 6 with diabetes, and 15 (9 aneurysm and 6 ectasia) abnormal group individuals. There was a very strong and significant correlation regarding the volume measurements between the two observers (r = 0.993, *p* < 0.001) (Fig. 2A). This relationship also held true regarding the total coronary volume (RCA, LAD, and LCX) of 29 patients between observers (r = 0.996, *p* < 0.001) (Fig. 2B). The absolute values produced by observer 1 versus observer 2 were very similar (867 ± 415 mm^3^ vs. 893 ± 486 mm^3^ for the diabetic group [n = 5], 1673 ± 494 mm^3^ vs. 1668 ± 486 mm^3^ for the normal group [n = 9], and 1644 ± 568 mm^3^ vs. 1647 ± 608 mm^3^ [n = 15] for the abnormal group). Furthermore, the intraclass correlation coefficient values for the measurements of LAD, LCX, and RCA were 0.963, 0.978, and 0.999, respectively, demonstrating excellent inter-observer measurement consistency (see Table S5 in supplementary material). Overall, this showed that there was no systematic error between observers and negligible skilled-operator bias.

## Discussion

To our knowledge, this work constitutes the first demonstration of a quantitative comparison of 3D coronary artery characteristics by coronary volume reconstruction. This evaluation was undertaken to test the feasibility and reproducibility of 3D QCA derived coronary volume analysis, in the first instance to assist with the challenging angiographic diagnosis of ectasia/aneurysm. Confirmation of a difference in coronary volume between a normal population and selected cohort of patients with angiographic ectasia or aneurysm is reassuring but not surprising. However, our segmental analysis has confirmed that despite increased vessel dimensions throughout all major epicardial coronary arterial segments, a significant increase in coronary volume is predominantly observed in the proximal segments. Importantly, low inter-observer variability was observed, however, automation of this enhanced QCA would further enhance the operator’s interpretation of the coronary angiogram and improve the diagnostic yield/identification of important non-stenotic coronary abnormalities.

Coronary artery aneurysm and ectasia are associated with a poor prognosis^9,10^. Previous studies have demonstrated that thrombosis and embolisation of the involved segments are the leading cause of acute MI in patients with coronary artery dilation^11,12^. In our previous report focusing on the abnormal cohort, despite low prevalence (3.4%) in patients under 50 years of age, 71.4% of all patients with coronary artery dilation presented with an acute ST-elevation myocardial infarction^3^. Furthermore, angiographic signs of turbulent and stagnant flow, have been demonstrated in patients with coronary artery dilation by using doppler velocity and thrombolysis in myocardial infarction (TIMI) frame count method, an index of coronary flow velocity^13,14^. However, at present, there is no data regarding an association between the coronary volume and coronary flow velocity. Our results are consistent with 3D coronary artery volume reconstructions of dilated coronary arteries being significantly larger than the volume of equivalent normal and diabetic arteries. In the future, use of 3D QCA may enable a better understanding of the association between coronary volume and flow dynamics in patients with dilated coronary arteries.

The inclusion of an additional cohort of diabetics with qualitatively normal coronary arteries was considered to test the new method of 3D QCA in the detection of possible early angiographic changes relating to diabetic vasculopathy. Hyperglycaemia has long been known as a risk factor in the progression of vascular complications of diabetes leading to changes in blood vessel diameter in arterial and arteriolar vessels^15,16^. Furthermore, increasing blood glucose may also have an effect on vasoconstriction^17^. As a consequence of this hyperglycaemic phenomenon, diabetes may be associated with CAD leading to MI and angina^18^. Indeed, Mosseri et al. showed that coronary arteries of diabetic patients had a smaller diameter than those of normal subjects^19^. In the present study, we observed that this is particularly apparent in the LAD (segment 6 and 7), and LCX (segment 11 and 13) with a trend towards smaller diameters in the RCA (see Table S3 in supplementary material). Furthermore, our measurements of total coronary artery volume of the diabetic group were significantly smaller than the normal group. This phenomenon might be explained by the effect of chronic hyperglycaemia which impairs functional vasodilation via increasing thromboxane-receptor-mediated vasoconstriction^20^.

Despite the pilot nature of our data, we have demonstrated a moderate correlation between total coronary volume and BSA. It has previously been demonstrated that there is a linear correlation between coronary artery diameter and BSA in children^21^. As a result, BSA-adjusted coronary artery Z-scores (standard deviations from the mean) have been used for classifying coronary artery dilation in young patients with Kawasaki disease^22^. However, as the paediatric Z-score is an ultrasound-based measurement and is not possible in adults, there is no diagnostic equivalent for adults with coronary aneurysm or ectasia. Beyond the choice of imaging modality, there are other important differences between the paediatric and adult populations. The adult population has a stable height but fluctuant body weight that may lead to a less consistent correlation between coronary volume and BSA, consequently indexing against other anthropomorphic parameters may prove more useful. Assessment of total coronary volume and height in our population provided a similarly moderate correlation (R = 0.42), which was partially enhanced when restricted to the normal group (R = 0.49). Correction of measurements to myocardial mass may provide the most accurate method of indexing, however, this would require multi-modality imaging. It is anticipated that extending our analysis to a larger cohort will further enhance our correlations and provide the potential for producing a Z-score equivalent using total coronary artery volume to differentiate pathological coronary artery dilation from healthy individuals.

Analysis of luminal diameters of normal coronary arteries are well established although differences in study methodology can make comparison challenging^23–25^. In the recent study by Medrano-Garcia et al., diameter and lengths of normal coronary artery were evaluated using computed tomography angiograms^26^. It is useful to compare our angiographically derived measurements against CT-derived measurements, albeit from separate populations. Our population had larger vessel diameters across all proximal segments, however, the derived LAD and LCX proximal segment lengths were shorter (22.6 ± 9.5 mm and 16.5 ± 8.3 mm, respectively vs. 35.5 ± 15.2 mm and 41.3 ± 20.4 mm by CT). Whereas, LMCA and RCA lengths appeared remarkably consistent (8.2 ± 4.4 mm and 106 ± 22.1 mm, respectively vs. 10.5 ± 5.3 mm and 106 ± 28.8 mm for CT). The variance in this comparison is likely to reflect the dependence of our 3D QCA analysis on optimally selected angiographic projections to generate an accurate reconstruction. Ultimately it would be useful to undertake a comparison of 3D QCA by multiple modalities to validate the coronary volume measurements.

There are several limitations that should be acknowledged. Firstly, this study was designed as a ‘proof-of-concept’ evaluation and consequently was not adequately powered to assess for significant differences between individual QCA parameters, or to provide sufficient data to generate a nomogram or thresholds for identification of coronary volume abnormalities. For this reason, angiographically evident coronary disease was excluded, however, we acknowledge that clinical application of this 3D QCA analysis will necessitate inclusion of a full spectrum of coronary disease. We anticipate that segmental-level analysis would facilitate comparative assessment in the presence of significant CAD. Secondly, as indicated by the comparison with CT-derived coronary dimensions, the retrospective nature of our analysis prevented optimisation of angiographic acquisition for each coronary segment. Of the initial population of 131 patients, 13 were excluded due to an inability to generate 3D reconstructions of all 3 coronary arteries. The majority of these patients had significant ectasia or aneurysm that significantly distorted image reconstruction. However, it is important to recognise that retrospective foreshortening with underestimation of coronary segment lengths is likely to contribute to the differences observed between modalities. Our intention is to repeat the analysis using a validation cohort from a collaborating centre and the ultimate goal is to undertake a prospective study utilising a dedicated acquisition/analysis protocol. Particular care will be taken to optimise image acquisition and minimisation of distortion of the 3D reconstruction of the coronaries, before considering our novel method as a clinical application for diagnosis of non-atherosclerotic coronary artery abnormalities.

Evaluation of coronary angiographic images tends to be qualitative in clinical practice, and consequently non-stenotic disease can be challenging to detect. We have demonstrated the feasibility of a 3D QCA methodology to quantify coronary volume in three distinct patient populations. This quantitative analysis correlates with BSA, has good reproducibility, and therefore offers potential as a tool for measuring coronary artery volume and identification of non-atherosclerotic coronary abnormalities. Larger scale, prospective, studies will be needed to generate adult Z-scoring for accurate identification and monitoring of abnormal coronary artery dilation, including aneurysm and ectasia.

## Methods

### Study population and definitions of each group

We have previously reported the prevalence of aneurysmal and ectatic disease within an unselected population of 1437 patients, under 50 years-of-age, that underwent coronary angiography between 2011 and 2015, at the Bristol Heart Institute, Bristol, UK^3^. We have extended the analysis to include two further populations from this cohort: patients found to have angiographically normal arteries, with or without a history of diabetes mellitus.

Patients were excluded if they had poor angiographic images or if there was only one angiographic view per artery, as neither are suitable for 3D QCA.

This study falls outside the scope of the UK policy framework for health and social care research and was registered with University Hospitals Bristol and Weston NHS Foundation Trust as a service evaluation. It is an analysis of routinely collected anonymized data, and followed the national “Guidance on the use of patient images obtained as part of standard care for teaching, training and research” issued by the Royal College of Radiologists, UK.

### Group with healthy coronary artery (normal group)

There were 344 patients with atypical chest pain who underwent coronary angiography to investigate for CAD, without evidence of any luminal stenosis/coronary atheroma. We excluded patients who had a history of myocardial infarction (MI), renal failure, diabetes, and > 2 risk factors for CAD, including hypertension, dyslipidaemia, smoking, and family history of CAD in first degree relatives less than 60 years of age, as these factors could affect coronary volume.

### Group with diabetic coronary arteries (diabetic group)

There were a total of 150 patients with insulin dependent diabetes investigated by coronary angiography for stable anginal symptoms. Insulin treatment was identified as as a surrogate for extended hypo-glycaemic treatment and thereby significant exposure to the potential vasculopathic effects of diabetes. Patients with evidence of luminal stenosis secondary to overt atherosclerosis were excluded from the analysis.

### Group with aneurysmal or ectatic coronary arteries (abnormal group)

All coronary angiograms demonstrating any discrepancy of vessel calibre were reviewed, independently, by two interventional cardiologists. Coronary aneurysm was defined by quantitative dilatation of the coronary artery exceeding 50% of an adjacent segment diameter and ectasia was dichotomized by evidence of extension across 2 or more coronary segments^27^.

### 3D volumetric coronary angiographic analysis

**Figure 3:**
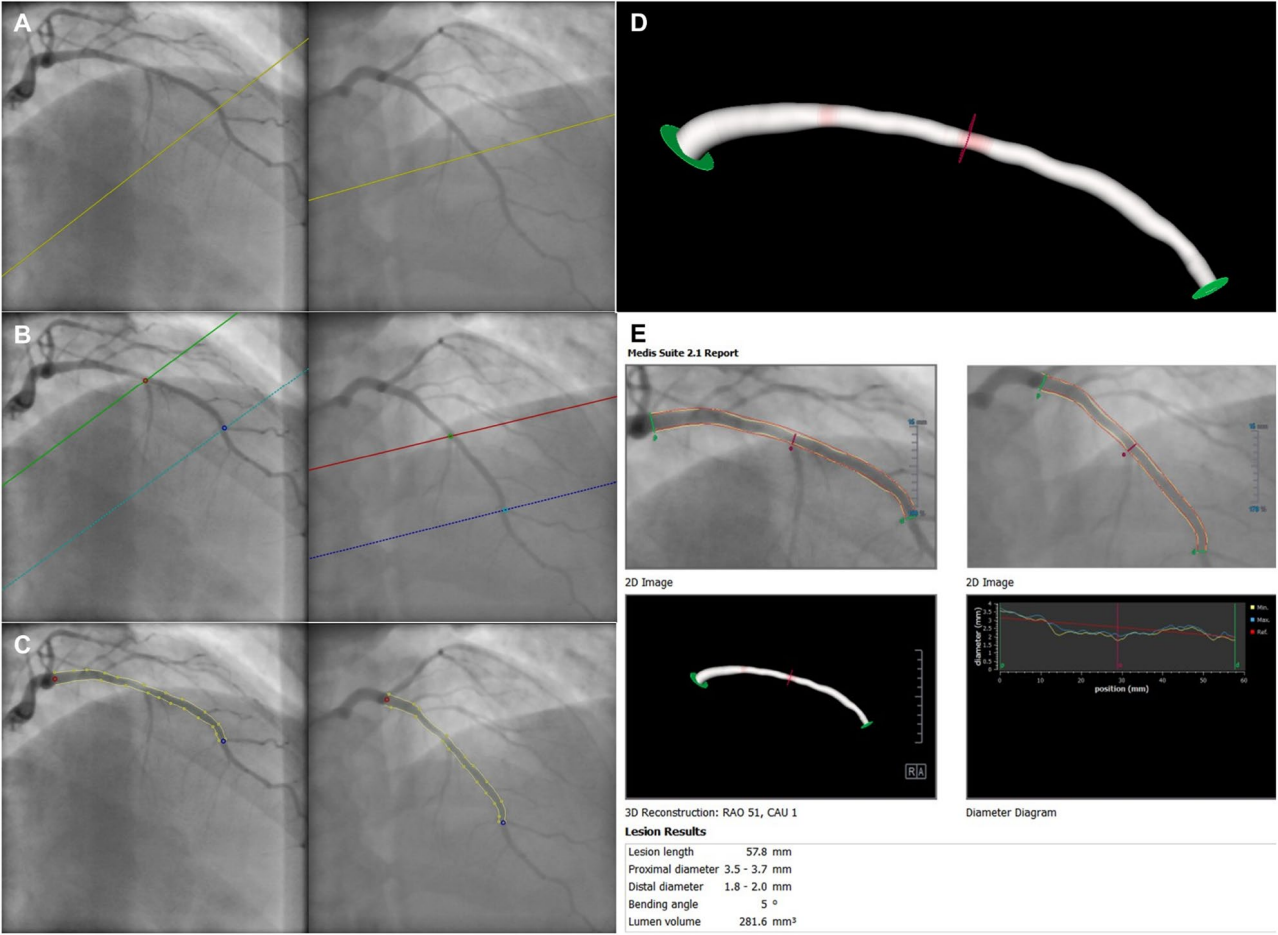
The process of 3D angiographic coronary artery volume reconstruction by 3D QCA. (**A**) Selection of the routine coronary angiography acquisition, two image sequences acquired at two angiographic views with projection angles at least 25° apart. (**B**) Identifying one to two anatomical markers such as bifurcations and side branches for automated correction of system distortions in the image geometry. (**C**) Manually defining the corresponding start and end positions of the interested vessel segment and extracting its contours and centerline. (**D**) Automating 3D coronary artery reconstruction of the vessel segment of interest. (**E**) Final 3D QCA report, including coronary volume, length, and proximal and distal diameter of the segment of interest. The diameter analysis includes a minimum (yellow line), maximum (blue line) and reference (red line) dimension. Abbreviations: *QCA* quantitative coronary angiography.

3D angiographic coronary artery reconstruction was performed using QAngio XA 3D (Research Edition, Medis medical imaging systems, Leiden, The Netherlands). The 3D reconstruction procedure consisted of the following steps: (1) from the routine coronary angiography acquisitions, two image sequences acquired from two angiographic projections at least 25° apart were highlighted; (2) end-diastolic still frames with complete vessel contrast-filling were selected (Fig. 3A); (3) one to two anatomical markers such as bifurcations and side branches were identified for automated correction of system distortions in the image geometry for the 3D angiographic reconstruction (Fig. 3B); (4) the corresponding start and end positions of the vessel segment of interest and its contours and centre line were semi-automatically defined (Fig. 3C); (5) automated 3D reconstruction was then performed (Fig. 3D). The data extract from the 3D analysis included lumen volume, proximal and distal diameters, and segment length (Fig. 3E).

### Coronary artery segments classification

Two interventional cardiologists determined each coronary segment according to the American Heart Association (AHA) classification^28^ (adapted from the original CASS segmentation model). If the AHA classification was not suitable for evaluating a segment, the segment would be defined using the modified AHA classification^29^. Volumetric analysis was restricted to the proximal and mid-vessel segments of all three major epicardial arterial territories. According to the segment classification, the right coronary artery (RCA) analysis included segments 1, 2, and 3. Regarding the left coronary system, the left main coronary artery (LMCA) segment 5, the left anterior descending artery (LAD) segments 6 and 7, and the left circumflex artery (LCX) segments 11 and 13 were included in the analysis.

### Statistical analysis

Continuous variables were expressed as mean with standard deviation (SD) and differences were investigated by use of one-way analysis of variance (ANOVA). Post-hoc analysis was conducted using ﻿Tukey Honest Significant Differences. Categorical variables were reported as numbers with percentage and compared with χ^2^ test. Correlations were assessed using the Pearson’s correlation coefficient, and the intraclass correlation coefficient was used to evaluate consistency of inter-observer agreement of coronary volume measurements. All variables were considered significant when the *p* value was < 0.05. Statistical analysis was performed using SPSS 22.0 for Windows (SPSS-PC, Chicago, IL, USA) and R version 3.6.0 (2019-04-26—R Core Team (2019). R: A language and environment for statistical computing. R Foundation for Statistical Computing, Vienna, Austria. URL https://www.R-project.org/.)

## Supplementary Information

Supplementary Tables.
