# Supplementary material for: Quantitative evaluation and comparison of coronary artery characteristics by 3D coronary volume reconstruction
Source: Sci Rep. 2021 Jan 13;11:1170. doi: 10.1038/s41598-020-80928-4 (PMC7806746; doi:10.1038/s41598-020-80928-4)
Supplement: Supplementary file 1 — Supplementary Tables. [file 41598_2020_80928_MOESM1_ESM.docx]

**Quantitative evaluation and comparison of coronary artery characteristics by 3D coronary volume reconstruction**

Yongcheol Kim ^a,b,1^, Jonathan J. H. Bray ^c,1^, Benjamin Waterhouse, Alex Gall ^b,1^,
Georgia Connolly ^b,1^,Eva Sammut ^b,1^, Vito D. Bruno ^b,1^, Robert Tulloh ^b,c,1^, David Adlam ^d,1^, Thomas W. Johnson ^b,^*^,1^

^a^ Division of Cardiology, Department of Internal Medicine, Yonsei University College of Medicine and Cardiovascular Center, Yongin Severance Hospital, Yongin, Republic of Korea; ^b^ Bristol Heart Institute, University Hospitals Bristol NHS Foundation Trust, UK; ^c^ University of Bristol, Bristol, UK; ^d^ Department of Cardiovascular Sciences, and NIHR Leicester Biomedical Research Centre, Leicester, UK

**Supplementary Material**

**Table S1:** Baseline characteristics in each group.

**Table S2:** Comparison of the length of coronary arteries in groups.

**Table S3:** Comparison of the lumen diameters between the groups.

**Table S4:** Comparison of coronary volumes/length ratio(mm^3^/mm)

**Table S5:** Intraclass correlation coefficient (ICC) values for individual arteries and total volume.

Table S1. Baseline characteristics in each group.

| Variables | All patients  (n=131) | DM group  (n=36) | Normal group  (n = 42) | Abnormal group  (n = 53) | P value |
| --- | --- | --- | --- | --- | --- |
| Age (years) | 43.2 (5.3) | 43.2 (5.8) | 41.9 (6.5) | 44.3(3.5) | 0.275 |
| Male sex | 100 (76.3%) | 22 (61.1%) | 28 (66.7%) | 50 (94.3%) †‡ | <0.01 |
| Rt. dominant artery | 98 (74.8%) | 25 (69.4%) | 32 (76.2%) | 41 (77.4%) | 0.679 |
| Risk factors for CAD | 2.0 (1.6) | 3.4 (1.1)* | 0.8 (1.2) | 2.0 (1.5) †‡ | <0.01 |
| Height (cm) | 172.8 (10.1) | 169.9 (9.0) | 173.3 (11.0) | 174.3 (10.0) | 0.058 |
| Weight (kg) | 89.6 (24.2) | 88.4 (20.3) | 81.7 (17.4) | 96.8 (29.1)† | 0.018 |
| BMI (kg/m^2^) | 30.0 (7.0) | 30.5 (5.7) | 27.3 (6.3) | 31.9 (7.8)† | <0.01 |
| BSA (m^2^) | 2.06 (0.30) | 2.03 (0.26) | 1.97 (0.23) | 2.15 (0.35)† | 0.043 |

Data are mean (SD) or n (%)

Abbreviation: BMI, body mass index; BSA, body surface area; CAD, coronary artery disease; DM, Diabetes Mellitus

†: Significant difference between abnormal vs normal

‡: significant difference between abnormal vs DM

*: significant difference between DM and normal

Table S2. Comparison of the length of coronary arteries in groups

|  | DM group | | Normal group | | Abnormal group | p value^§^ |
| --- | --- | --- | --- | --- | --- | --- |
|  | Length (mm) | Length (mm) | | Length (mm) | |  |
| LAD | 52.4 (12.5) | 55 (16.8) | | 52.3 (15.1) | | 0.581 |
| pLAD | 19.3 (8.4) | 22.6 (9.5) | | 22.1 (10.2) | | 0.251 |
| mLAD | 33.2 (12.4) | 32.4 (14.2) | | 30.2 (12.2) | | 0.607 |
| LCX | 39.7 (14.3) | 46.6 (12.5) | | 48.1 (15.4) | | 0.027 |
| pLCX | 17.5 (8.8) | 16.5 (8.3) | | 18.7 (10.6) | | 0.287 |
| mLCX | 22.5 (13.1) | 29.7 (12.5) | | 29.5 (13.7) | | 0.033 |
| RCA | 98.0 (17.9) | 106 (22.1) | | 106.6 (20.2) | | 0.232 |
| pRCA | 30.6 (10.4) ^‡^ | 35.8 (13) | | 38.4 (9.88)^‡^ | | 0.015 |
| mRCA | 23.5 (9.4) | 28.5 (13.6) | | 25.7 (12.5) | | 0.287 |
| dRCA | 43.8 (14.1) | 41.9 (15.6) | | 42.2 (16.5) | | 0.741 |
| LMCA | 10.9 (4.6)^*^ | 8.2 (4.4) ^*^ | | 9.7 (4.04) | | 0.022 |

Data are expressed as mean (SD).

^§^P value for length

* significant DM vs Normal

† significant Normal vs abnormal

‡ significant abnormal vs DM

Abbreviation: p-, m-LAD, proximal-, mid-left anterior descending artery; p-, m-LCX, proximal-, mid-left circumflex artery; p-, m-, d-RCA, proximal- mid- distal-right coronary artery; LMCA, left main coronary artery; DM, Diabetes Mellitus

Table S3. Comparison of the lumen diameters between the groups

|  | DM group | | | Normal group | | | | Abnormal group | | | |
| --- | --- | --- | --- | --- | --- | --- | --- | --- | --- | --- | --- |
|  | aDia (mm) | pDia (mm) | dDia (mm) | | aDia (mm) | pDia (mm) | dDia (mm) | | aDia (mm) | pDia (mm) | dDia (mm) |
| LAD | 2.6 (0.5)^*^ | 3.3 (0.6) | 2.0 (0.4)* | | 3.0 (0.4)^†^ | 3.5 (0.5)† | 2.4 (0.5) | | 3.3 (0.6)^‡^ | 4.6 (1.2)‡ | 2.4 (0.5)‡ |
| pLAD | 3.0 (0.6)^*^ | 3.3 (0.6) | 2.7 (0.7) | | 3.4 (0.4)^†^ | 3.5 (0.5)† | 3.0 (0.5) | | 3.9 (0.8)^‡^ | 4.5 (1.2)‡ | 3.3 (0.8)‡ |
| mLAD | 2.3 (0.5)^*^ | 2.7 (0.7) | 2.0 (0.4)* | | 2.7 (0.5)^†^ | 3.0 (0.5) | 2.4 (0.5) | | 2.8 (0.6)^‡^ | 3.3 (0.8)‡ | 2.4 (0.5)‡ |
| LCX | 2.4 (0.6)^*^ | 2.9 (0.6) | 1.9 (0.6)* | | 2.8 (0.4) | 3.2(0.5)† | 2.4 (0.6) | | 3.0 (0.6)^†^ | 3.9 (1.2)‡ | 2.3 (0.7)‡ |
| pLCX | 2.7 (0.6)^*^ | 2.9 (0.6) | 2.4 (0.7)* | | 3.1 (0.4)^†^ | 3.2 (0.5)† | 2.9 (0.4) | | 3.5 (0.7)^‡^ | 3.9 (1.2)‡ | 3.2 (0.7)‡ |
| mLCX | 2.2 (0.6)^*^ | 2.5 (0.7)* | 1.9 (0.6)* | | 2.6 (0.5) | 2.9 (0.4) | 2.4 (0.6) | | 2.7 (0.6)^‡^ | 3.2 (0.7)‡ | 2.3 (0.6)‡ |
| RCA | 2.7 (0.6) | 3.2 (0.7) | 2.1 (0.6) | | 3.0 (0.5) | 3.2 (0.6)† | 2.3 (0.5) | | 3.4 (0.9)^‡^ | 3.7 (0.8)‡ | 2.3 (0.7) |
| pRCA | 3.0 (0.6) | 3.2 (0.7) | 2.7 (0.7)* | | 3.4 (0.6) | 3.2 (0.6)† | 3.2 (0.6) | | 3.7 (0.8)^‡^ | 3.6 (0.8)‡ | 3.4 (0.8)‡ |
| mRCA | 2.6 (0.7)^*^ | 2.7 (0.6)* | 2.4 (0.7)* | | 3.0 (0.6) | 3.2 (0.6) | 2.9 (0.5) | | 3.3 (0.9)^‡^ | 3.5 (0.9)‡ | 3.1 (0.9)‡ |
| dRCA | 2.4 (0.7) | 2.4 (0.7)* | 2.1 (0.6) | | 2.7 (0.6) | 2.9 (0.5) | 2.3 (0.5) | | 2.8 (0.8)^‡^ | 3.1 (0.9)‡ | 2.3 (0.8) |
| LMCA | 4.0 (0.6) | 4.0 (0.7) | 3.9 (0.6) | | 4.2 (0.6)† | 4.0 (0.7)† | 4.1 (0.6)† | | 4.8 (0.8)^‡^ | 4.8 (0.8)‡ | 4.8 (0.9)‡ |

Data are expressed as mean (SD).

* significant DM vs Normal

† significant Normal vs abnormal

‡ significant abnormal vs DM

Abbreviation: LAD, left anterior descending artery; LCX, left circumflex artery; RCA, right coronary artery; LMCA, left main coronary artery; aDia, average lumen diameter; pDia, proximal lumen diameter; dDia, distal lumen diameter; DM, Diabetes Mellitus

Table S4. Comparison of coronary volumes/length ratio(mm^3^/mm)

|  | DM group (n = 36) | Normal group (n = 40) | Abnormal group (n = 42) | P value^§^ |
| --- | --- | --- | --- | --- |
|  | Volume/length (mm^3^/mm) | Volume/length (mm^3^/mm) | Volume/length (mm^3^/mm) |  |
| LAD | 5.30 (1.90)^*^ | 7.32 (2.03)^*,†^ | 8.91 (2.89)^†^ | < 0.001 |
| Proximal LAD | 7.21 (2.95)^‡^ | 9.07 (1.97)^†^ | 12.70 (5.04)^†‡^ | < 0.001 |
| Mid LAD | 4.29 (1.87)^*^ | 6.12 (2.23)^*,†^ | 6.69 (3.10)^†^ | <0.001 |
| LCx | 4.94 (2.32)^*^ | 6.33 (1.83)^*,†^ | 7.84 (3.13)^†^ | < 0.001 |
| Proximal LCx | 5.83 (2.6)^‡,*^ | 7.71(1.96)^*†^ | 9.84 (4.15)^†‡^ | < 0.001 |
| Mid LCx | 4.1 (2.33)^*^ | 5.53 (1.84)^*,†^ | 6.14 (2.98)^†^ | 0.002 |
| RCA | 5.91 (2.72)^‡^ | 7.44 (2.42) | 8.98 (3.77)^‡^ | < 0.001 |
| Proximal RCA | 7.6 (2.99)^‡^ | 9.15 (2.74) | 11.39 (4.93)^‡^ | < 0.001 |
| Mid RCA | 5.82 (3.07)^‡^ | 7.47 (2.43) | 9.06 (4.67)^‡^ | 0.001 |
| Distal RCA | 4.78 (2.48)^‡^ | 5.87 (2.28) | 6.8 (3.43)^‡^ | 0.008 |
| LMCA | 12.54 (3.29)^‡^ | 14 (3.52)^†^ | 18.43 (5.89)^†‡^ | <0.001 |

Data are expressed as mean (SD).

^§^ANOVA for DM group vs. Normal group vs. Abnormal group.

^*, †, ‡^ The corresponding values with different symbols are significantly different from each other.

Abbreviation: LAD, left anterior descending artery; LCx, left circumflex artery; RCA, right coronary artery; LMCA, left main coronary artery; DM, Diabetes Mellitus

Table. S5. Intraclass correlation coefficient (ICC) values for individual arteries and total volume.

| Measure | ICC | p value |
| --- | --- | --- |
| LAD volume (n = 29) | 0.963 | < 0.001 |
| LCX volume (n = 30) | 0.978 | < 0.001 |
| RCA volume (n = 30) | 0.999 | < 0.001 |
| Total volume (n = 29) | 0.996 | < 0.001 |

LAD = segments 6 and 7; LCX = segments 11 and 13; RCA = segments 1, 2, and 3.

Abbreviation: LAD, left anterior descending artery; LCX, left circumflex artery; RCA, right coronary artery
